# *Adansonia digitata* germination tests. Elephants or heat: what causes scarification of seed to facilitate germination?

**DOI:** 10.1186/s40529-020-00296-0

**Published:** 2020-06-16

**Authors:** Thea Lautenschläger, Nele Teutloff, Markus Günther, Christoph Neinhuis

**Affiliations:** Department of Biology, Institute of Botany, Faculty of Science, Technische Universität Dresden, Dresden, 01062 Germany

**Keywords:** Seed dormancy, Seed coat, Acid, Digestion, Heat, Fire, Soil temperature, Solar radiation, Scarification

## Abstract

**Background:**

The dormancy of *Adansonia digitata* seeds is well known. For propagation purposes, plenty of germination tests were conducted, however, rarely taking the ecology of baobab into account. Our main goal, therefore, is to identify the decisive natural trigger for breaking the dormancy. We therefore performed 31 different tests and their influence on the germination rate (time to germination and proportion of seeds germinating).

**Results:**

The highest germination rates were reached in the heat tests while elephant’s digestion seems to stimulate germination of *Adansonia digitata* only to a limited extent. The chalazal slit of the seed represents the primary site of water entry. Tannins concentrated in this region that are influenced by temperature play an important role for inhibiting the germination.

**Conclusion:**

As a result, the hypothesis is formulated that germination success strongly depends on heat, provoked by wildfires or prolonged exposition to the sun causing decomposition of tannins by high temperatures rather than on digestion.

## Introduction

Seeds of *Adansonia digitata* L. (Malvaceae) possess a thick and firm testa. Protective tissues of large seeds are known to increase survival in fire-prone savanna (Gashaw and Michelsen 2002) but at the same time impede germination under normal conditions. The dormancy of *A. digitata* seeds released from ripe fruits imposed by their hard seed coats is well known (Niang et al. 2015) as seeds exhibit water-impermeable coats (Razanameharizaka et al. 2006) and a high amount of tannin between the chalazal cap and the embryo was described (Rao 1954). Several authors carried out germination tests on *A. digitata* seeds for conservation purposes (Esenowo 1991; Johansson 1999; Razanameharizaka et al. 2006; Niang et al. 2015; El-bably and Rashed 2018). To this effect, different test set-ups were used to allow scarification imitating digestion or heat processes. On the one hand, it is to be presumed that the passage through the digestive tract improves germination because it softens the testa and enhances water absorption (Chevalier 1906; Gebauer El-Siddig and Ebert 2002; McGrew et al. 2003), but for *A. digitata* reliable information is still lacking (Wickens and Lowe 2008). However, it was also found that the fruit pulp acts as a germination inhibitor (Esenowo 1991) so that it seems quite reasonable to assume that digestion could have a positive effect.

Regarding the impact of heat, several studies included a seed treatment in cold, hot or even boiling water (Esenowo, 1991; Razanameharizaka et al. 2006; Niang et al. 2015) but only Johansson (1999) studied the influence of dry heat on germination applying temperatures of 100 °C and 250 °C only. In this study, we therefore focused on a variety of heat treatments, which most probably occur in baobabs natural habitat. We wanted to clarify whether scarification of seed testa is more favourable using “digestive” methods or by heat impact. Heat treatments comprised not only wet but also dry heat at different temperatures to identify the range resulting in the highest germination rate and then contextualize the data to its ecological equivalent such as solar radiation, heating up the soil or heat caused by wildfires. Furthermore, we state different explanation attempts regarding the opening process during germination of the *Adansonia* seed because former studies on water gaps in seeds consider only anatomical details without taking into account the various factors inhibiting germination (Rao 1954; Corner 1976; Gama-Arachchige et al. 2013).

## Materials and methods

Six germination test sets were conducted, two in 2017 with 2 month of temporal distance, one test in 2018, two in 2019 and one in 2020. For every test set, seeds taken from ten fruits of *A. digitata* were used. These fruits were bought at the local central market in Uíge in northern Angola (S 7° 36’ 48’’, E 15° 03’ 27’’) in March 2016, February 2018 and April 2019.

All test sets differed from each other and were derived from previous experiments (Esenowo 1991; Johansson 1999; Razanameharizaka et al. 2006). While in 2017 only five variables for each attempt were tested (elephants´ digestion, 70 °C hot water for 40 min, 3 days cold water, 10 min in H_2_SO_4_, and no treatment) the test sets in 2018, 2019 and 2020 were expanded considerably.

To allow scarification by the digestive system of predators, fruits were opened and the fruit pulp with the embedded seeds was fed to three elephants (*Loxodonta africana*) in the Zoological Garden Dresden. Twenty-eight to 36 h later, seeds were picked out of the dung and concurrently sowed. In the 2018 test set, seeds were additionally sowed in the dung in which they were embedded. In 2019, elephant’s digestion and heat treatment were combined by picking the seeds from the dung subsequently exposing them to heat of 75 °C , 100 °C , 125 °C and 150 °C for 5 min. As chemical acids are often mentioned to imitate this intestinal scarification process, we also included this treatment by soaking the seeds in concentrated H_2_SO_4_ (pH 1,73) for 10 min in 2017 or rather 5, 10 and 15 min in 2018 with subsequent rinsing in running water for 3 h. We decided to use this method because Esenowo (1991) achieved the best results using H_2_SO_4_. Furthermore, we treated seeds with HCl of pH 2 and 3 for 1 or 2 h in 2019 as it most likely mimics gastric juice, which exhibits a pH value of 2–3 (Van Howen, Prins and Lankhorst 1981; Prenzlin 2009).

To detect the influence of heat, seeds were put into 70 °C hot water for 40 min, using a thermos flask to keep temperature constant. This temperature and time of exposure was recommended by Esenowo (1991). In the amplified test set, we included dry and moist heat with varying temperatures using a stove (AEG Competence PNC 944). For the dry heat, seeds were exposed to heat of 50 °C, 75 °C, 150 °C and 125 °C for 40 min each, whereas for the moist conditions seeds were imbedded in wet sand and then put into the oven. We expanded the test set in 2019 with dry heat of 75 °C, 100 °C, 125 °C and 150 °C for 5 min. These selected temperatures as well as the exposure time was adapted from Kempe et al. (2018) orienting to several studies on wildfire intensities (Trollope 1984; Miranda et al. 1993; Savadogo et al. 2007). One control was done with seeds put in cold water (20 °C) for 3 days (Johansson 1999), one negative control without any treatment, and additionally one control without treatment but sowed in the Botanic Garden in Uíge, Angola to verify *A. digitata* germination results within the natural range of the tree. As a positive control, we incorporated a manual nicking treatment (Table 1). Derived from Razanameharizaka et al. (2006), we carried out these germination tests according to ISTA (International Seed Testing Association 1999). Therefore, small parts of the seed coat (5–10 mm^2^) were first removed using pruning shears and then sowed in sterilized sand, moistened with distilled water. The germination boxes were stored at 30 ± 1 °C in a dark atmosphere of an incubator (Sanyo Incubator MIR-253) for 25 days. We considered a seed germinated if the radicle emerged through the seed coat.

**Table 1:** Control test setups: Relative germination rate of *Adansonia digitata* seeds after 10 weeks [%]

Treatment	2017a	2017b	2018	2019a/2020	Average
Negative control: no treatment	1	0	0	3	1
No treatment, planted in sun in Angola		0		13	13**
No treatment, planted in shadow in Angola				0	0
Positive control: manual nicking treatment				74	74**

Results obtained in three test setups with 100 seeds per treatment each: 2017a, 2017b, 2018 and 2019a. Significant increase of GR compared to the control without treatment are indicated with *P<0.05, **P<0.01

One hundred seeds of each experimental variant were used excepted for the combination of elephant’s digestion and heat treatment where the picked seeds in faeces just sufficed for 50 seeds per experiment. Apart from the elephant variant, fruit pulp was removed from all seeds by rubbing the seeds between hands because the pulp was proven to impede germination (Esenowo 1991; Johansson 1999). Thus, in total five variants each in two settings with in total 1.000 seeds in 2017, 16 variants with in total 1.600 seeds in 2018, 17 variants with 100 seeds, 4 variants with 50 seeds with in total 1.900 seeds in 2019 and 100 seeds in 2020 were used for germination tests.

Cultivation took place in a greenhouse of the Botanical Garden of the TU Dresden (average temperature of 21 °C and an average humidity of 82%) from the beginning of February to the end of September 2017, from beginning of March to end of end of June 2018 and from mid-February to the end of September 2019, respectively. According to sunrise and sunset, day lengths vary from 9 h in February and September to 16.5 h in June (http://www.sunrise-set.com). Seeds were irrigated every day. Successful germination was detected every day, except on weekends. Germination rates were clustered in 2-week periods. Recording of the germination success focussed on the first 10 weeks since we observed in 2017 that the influence of the treatments was noticeable only in the first 8 weeks, while later germination occurred only randomly (maximum 1 of 100 seeds per 4 weeks). This was proven by statistical analysis via student´s t-test (McDonald, 2015). Gashaw and Michelsen (2002) recorded germination rates of different Ethiopian woody savanna plant seeds after heat treatment for 9 weeks only, because thereafter no additional seedlings emerged while Razanameharizaka et al. (2006) documented germination rate only for 25 days. At the end of the experiment, germination counts were calculated in percentages (%). We checked whether treated seeds germinated significantly better than seeds without treatment using the Chi square-test of independence (McDonald 2015).

Furthermore, for seed morphometric comparisons with other germination studies, length of 20 seeds and 100-seed dry weight was documented. For seed length, a digital calliper with an accuracy of 0.1 mm was used. Weight was measured using an electronic balance (maximum = 81 g/200 g, d=0.01 mg/0.1 mg, model Mettler Toledo XA 205 DualRange). A scanning electron microscope (SEM; Zeiss Supra 40VP, Ulm, Germany) was used for studying the seed coat. The X-ray computerized tomography was equipped with a micro-focus tube and a CCD sensor taking pictures at 60 kV and a current of 200 µA.

## Results

### Germination tests

Scarification processes are important factors influencing germination rate (GR) as already proven earlier (Esenowo 1991; Razanameharizaka et al. 2006; Niang et al. 2015). As expected, seeds that were sowed without any treatment did not germinate, which again confirmed earlier results and can be explained by the dormancy of *Adansonia digitata* seeds and their thick testa of about 700 µm (Fig. 2a).

The test “digestion by elephants” only reached an average GR of 2.7% that still resulted in a significant increase of the GR (P<0.05). Whether seeds were sown in soil or in elephant’s faeces did not make a difference. Although the treatment with concentrated H_2_SO_4_ for 5, 10 and 15 min showed an increased GR with a maximum of 14% after 15 min exposure time these data are in strong contrast to the data obtained by Esenowo (1991) who reported a GR of 98% after 15 min in H_2_SO_4_. The treatment with HCL pH=2 as well as pH=3 for 1 or 2 h also confirm a low GR generated through acid treatment (0–2%). Seeds digested by an elephant before heat treatment show a significantly lower GR at temperatures of 75 °C and 100 °C for 5 min than seeds only treated with heat (P<0.01). The germination rates of the acid treatments are shown in Table 2.

**Table 2:** Acid treatment: relative germination rate of *Adansonia digitata* seeds after 10 weeks [%]

Treatment	2017a	2017b	2018	2019a	Average
H_2_SO_4_ for 5 min			0		0
H_2_SO_4_ for 10 min	12	7	3		7.3**
H_2_SO_4_ for 15 min			14		14**
HCl for 1 h, pH 2				0	0
HCl for 1 h, pH 3				2	2
HCl for 2 h, pH 2				1	1
HCl for 2 h, pH 3				2	2
Elephants´ digestion, germinating in soil	0	7	1		2.7*
Elephants´ digestion, germinating in elephants´ faeces			1		1

Results obtained in three test setups with 100 seeds per treatment each: 2017a, 2017b, 2018 and 2019a. Significant increase of GR compared to the control without treatment are indicated with *P<0.05, **P<0.01

Due to these results, we subsequently concentrated on heat impact and wanted to know whether higher temperatures may be responsible for the breaking seed dormancy and, if so, what the relevant temperature range may be. Heat treatment for a short period of time like 5 min shows a really high GR for 75 °C and 100 °C with a maximum of 78% at 100 °C. Apparently, the temperature is high enough to break the seed dormancy efficiently and the time short enough not to kill the embryo. While heat treatment under dry conditions above 100 °C for 40 min did not show any germination, presumably because the embryo was killed, temperatures ≤75 °C revealed significantly higher GR as compared to the control. 50 °C resulted in just a small but significant increase by 13% GR (P<0.01), while GR at 75 °C raised to 64% GR (P<0.01). The seeds treated under wet conditions (wet sand) germinated at even higher rates: 75 °C resulted in 72% GR. But again 100 °C most probably killed the embryos and no germination was observed. The germination rates of the different heat treatments are shown in Table 3. However, not only temperature determinate a border for breaking seed dormancy, also time of heat treatment seems to play an important role. Interestingly, the temporal progressions of germination differed between dry and wet heat. While the majority of seeds treated with hot water for 40 min or in wet hot sand germinated in the 3rd and 4th week after the treatment, those treated under dry conditions germinated 2 weeks later on average.

**Table 3:** Heat treatment: Relative germination rate of *Adansonia digitata* seeds after 10 weeks [%]

Treatment	2017a	2017b	2018	2019b/2020	Average
Cold water for 3 days	0	1	0		0.3
In hot water for 40 min 70°C	70	73	42	63	62**
Short-time heat treatment					
In oven for 5 min 75°C				51	51**
In oven for 5 min 100°C				78	78**
In oven for 5 min 125°C				11	11**
In oven for 5 min 150°C				3	3
Long-time heat treatment					
In oven for 40 min 50°C			13		13**
In oven for 40 min 75°C			64		64**
In oven for 40 min 100°C			0		0
In oven for 40 min 125°C			0		0
In wet sand in oven for 40 min 50°C			8		8**
In wet sand in oven for 40 min 75°C			72		72**
In wet sand in oven for 40 min 100°C			0		0
In wet sand in oven for 40 min 125°C			0		0
Combination of elephants´ digestion and heat treatment					
Elephants´ digestion + in oven 5 min 75°C				24	24**
Elephants´ digestion + in oven 5 min 100°C				28	28**
Elephants´ digestion + in oven 5 min 125°C				16	16**
Elephants´ digestion + in oven 5 min 150°C				4	4*

Results obtained in three test setups with 100 seeds per treatment each, except 50 seeds for combination of elephant’s digestion and heat treatment: 2017a, 2017b, 2018 and 2019b. Significant increase of GR compared to the control without treatment are indicated with *P<0.05, **P<0.01

Soaking the seeds during 3 days in cold water did not increase GR although Johansson (1999) stated that soaking in cold water for 3 days would break seed dormancy of *A. digitata* without further treatment. Nevertheless, with a maximum of 60% the GR in her experiments were comparatively low.

### Temporal progress of germination

**Fig. 1:**
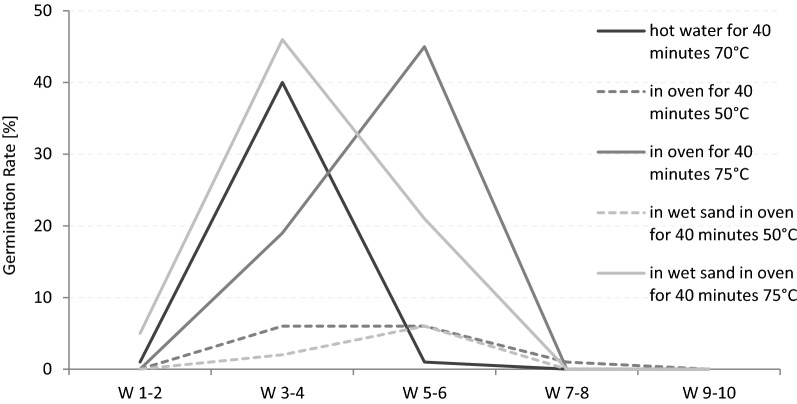
*Adansonia digitata* seed-germination during 10 weeks (W) after heat treatment. Blue: 70 °C hot water for 40 min. Red: Dry conditions in oven for 40 min. Orange: Wet conditions in the oven for 40 min. Dashed lines: 50 °C, solid lines: 75 °C

The student´s t-test showed that after 8 weeks significantly less germination occurs than during the first eight weeks (2017a: t=1.58, d.f.4, P=0.18; 2017b: t=1.0, d.f.4, P=0.37). Within that time, several periods of germination were observed, depending on seed treatment (Tables 4 and 5). In general, GR during the first 2 weeks and after the 6th week is low. Most of seeds treated with H_2_SO_4_, HCl or digested by elephants germinated within the third and 4th week. Looking at the heat treatments, the germination behaviour differs as follows: after treatments in hot water or wet hot sand for 40 min rapid germination occurred in week 3 and 4 while it was delayed by 2 weeks after exposure to dry hot sand (Fig. 1). In 2018, 95% of the germinated seeds treated with hot water germinated in week 3–4, 64% of those treated with wet sand (75 °C), but just 30% of the seed treated in dry heat (75 °C). The latter showed the main germination peak in week 5–6 (70%). At lower temperature (50 °C) this trend slows down but is still significant (P<0.01). The temporal progress of the different tests is shown in the Additional file 1.

**Table 4:** Acid treatment: Relative germination rate of *Adansonia digitata* seeds in percentage

Treatment	0–2 weeks	3–4 weeks	5–6 weeks	7–8 weeks	9–10 weeks
H_2_SO_4_ for 5 min	0	0	0	0	0
H_2_SO_4_ for 10 min	0.3^a^	5.7^a^	1^a^	0^a^	0.3^a^
H_2_SO_4_ for 15 min	2	12	0	0	0
HCl for 1 h, pH 2	0	0	0	0	0
HCl for 1 h, pH 3	0	0	1	1	0
HCl for 2 h, pH 2	0	1	0	0	0
HCl for 2 h, pH 3	0	2	0	0	0
Elephants´ digestion, germinating in soil	0.7^a^	1.7^a^	0^a^	0.3^a^	0^a^
Elephants´ digestion, germinating in elephants´ faeces	0	1	0	0	0

Numbers marked with ^a^are the average of the germination tests (2017, 2018, 2019) the remaining numbers are obtained in the test sets in 2018 or 2019

**Table 5:** Heat treatment: Relative germination rate of *Adansonia digitata* seeds in percentage

Treatment	0–2 weeks	3–4 weeks	5–6 weeks	7–8 weeks	9–10 weeks
Short-time heat treatment					
In oven for 5 min 75°C	0	26	21	4	0
In oven for 5 min 100°C	0	35	42	1	0
In oven for 5 min 125°C	0	5	4	2	0
In oven for 5 min 150°C	0	0	3	0	0
Long-time heat treatment					
In oven for 40 min 50°C	0	6	6	1	0
In oven for 40 min 75°C	0	19	45	0	0
In oven for 40 min 100°C	0	0	0	0	0
In oven for 40 min 125°C	0	0	0	0	0
In wet sand in oven for 40 min 50°C	0	2	6	0	0
In wet sand in oven for 40 min 75°C	5	46	21	0	0
In wet sand in oven for 40 min 100°C	0	0	0	0	0
In wet sand in oven for 40 min 125°C	0	0	0	0	0
Combination of elephants´ digestion and heat treatment					
Elephants´ digestion + in oven 5 min 75°C	2	12	10	0	0
Elephants´ digestion + in oven 5 min 100°C	0	18	10	0	0
Elephants´ digestion + in oven 5 min 125°C	0	8	8	0	0
Elephants´ digestion + in oven 5 min 150°C	2	2	0	0	0

Numbers marked with ^a^are the average of the germination tests (2017, 2018, 2019) the remaining numbers are obtained in the test sets in 2018 or 2019

### Seed morphometrics

The average length of *A. digitata* seeds collected in northern Angola is 12.0 ± 0.39 mm. The 100-seed dry weight is 53.75 g.

### Visualization of testa and germination process

**Fig. 2:**
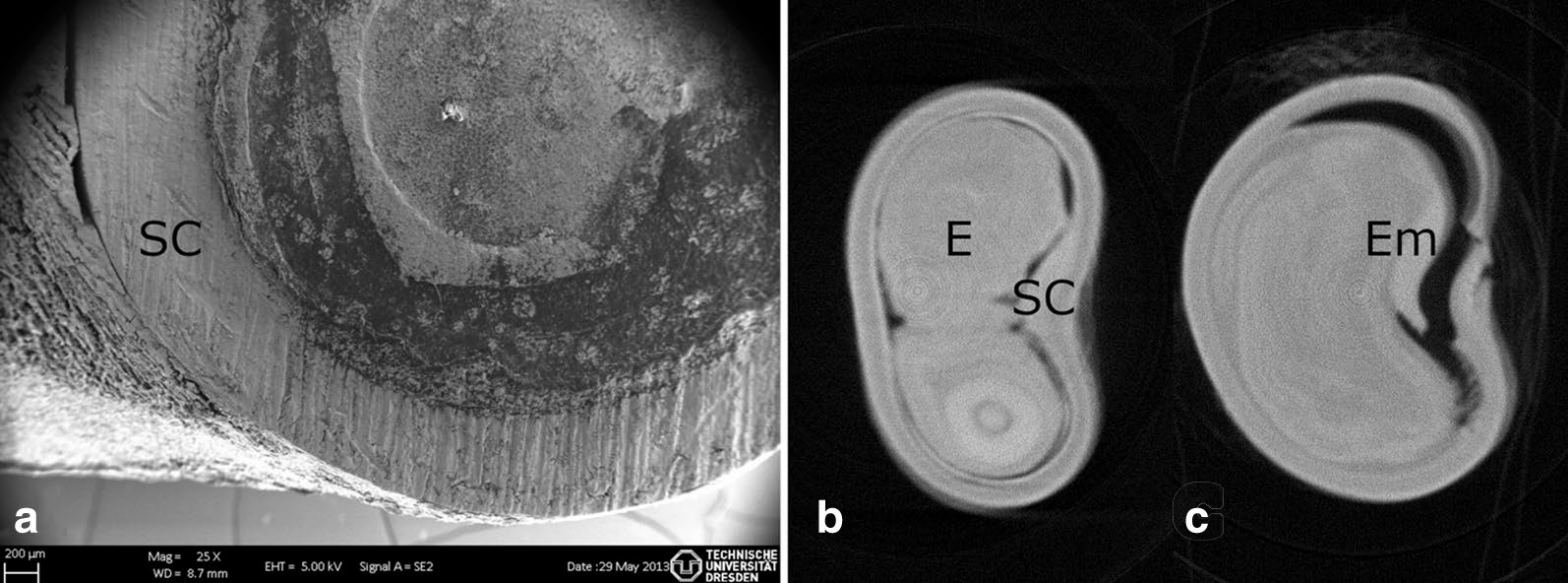
Seed of *Adansonia digitata*. **a** SEM-picture of the firm testa with a thickness of 700 µm, *E* endosperm, *Em* embryo, *SC* seed coat; (**b**) and (**c**) tomography in lateral view: (**b**) dry untreated seed; (**c**) seed after immersion in water at 100 °C. Due to the swelling cavities disappear and seed coat cracks open on the inside of the reniform seed

**Fig. 3:**
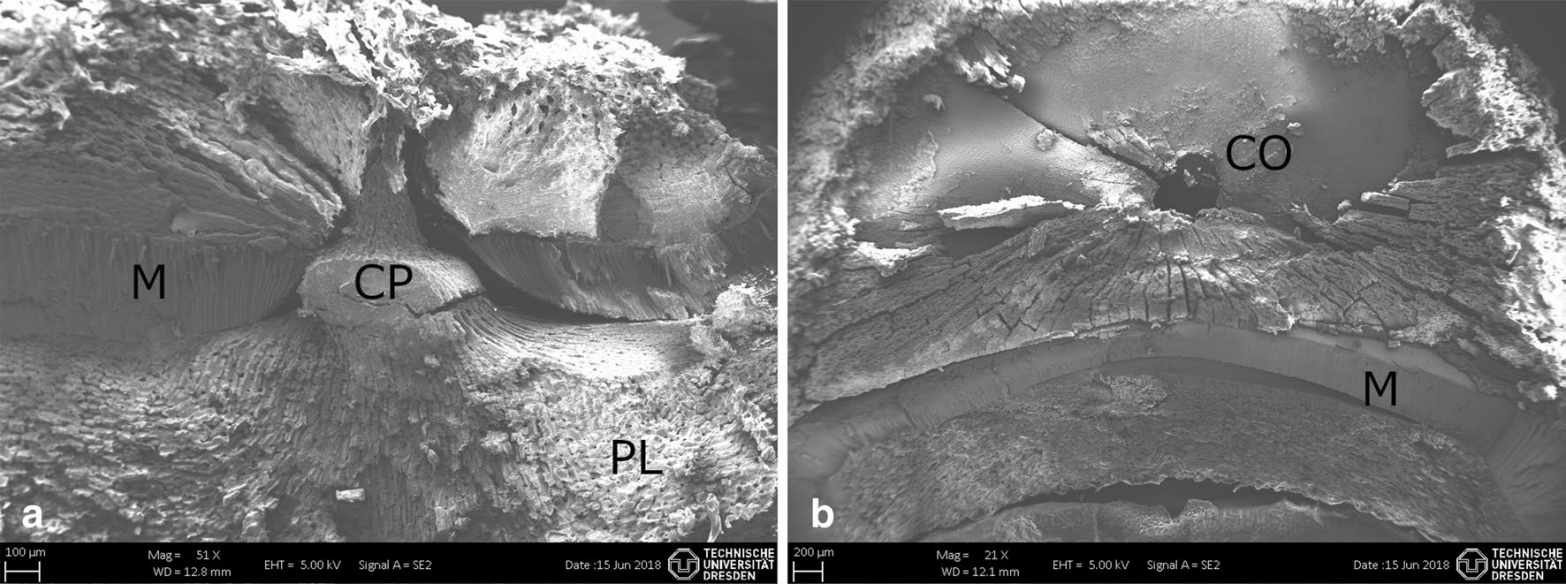
SEM-pictures of germination mechanism of *Adansonia digitata* seed at the chalaza, *CO* chalazal oculus, *CP* chalazal plug, *M* Malpighian cells, *PL* inner pigment layer with tannin. **a** side view of the chalazal plug; **b** top view on the chalazal oculus after removing the plug

**Fig. 4:**
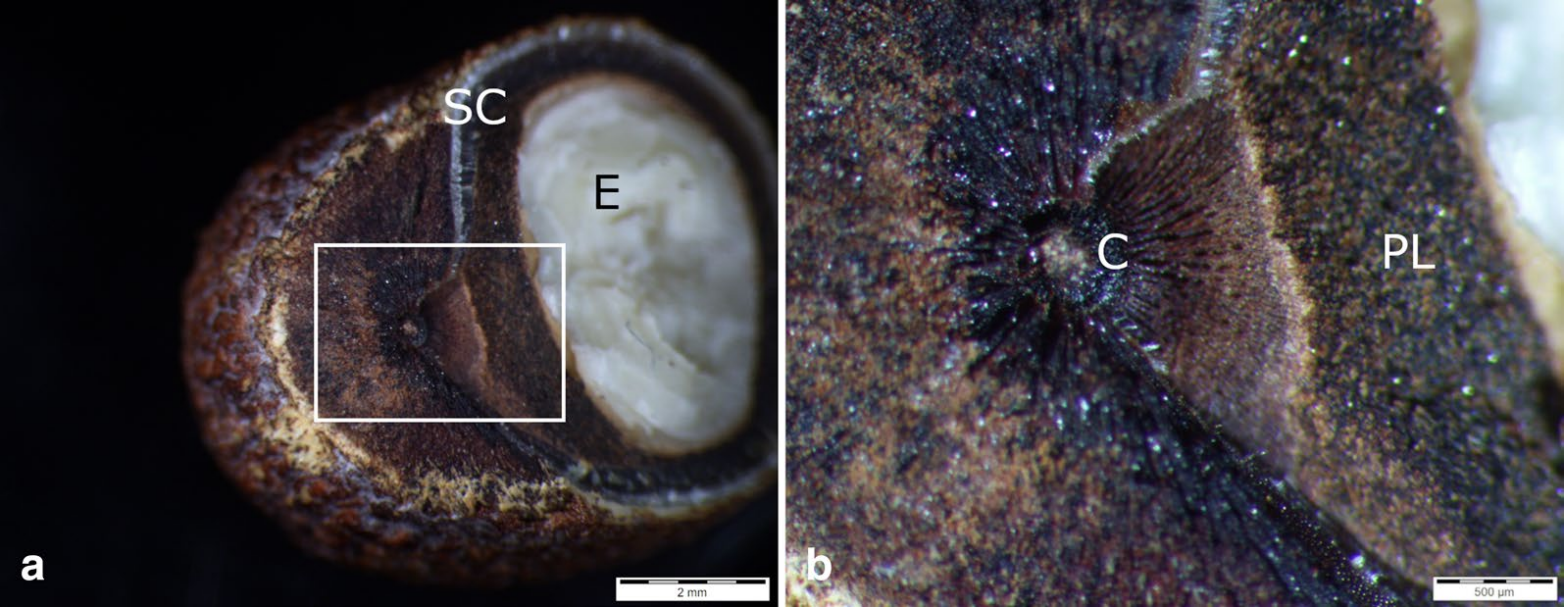
Incident-light microscopy images of *Adansonia digitata* seed, at the inner surface of the reniform seed, *C* chalaza, *E* endosperm, *PL* inner pigment layer with tannin, *SC* seed coat. **a** Seed fracture to bare the chalaza; **b** close-up view of the chalaza structure

The seed coat of *Adansonia digitata* seeds is shown in Fig. 2a. It is composed of the 6–8 celled testa, and the tegmen whose exotegmen consists of exceptionally long Malpighian cells with a distinct light line forming an up to 1 mm high palisade layer (Rao 1954; Corner 1976). Furthermore, an inner pigment layer exhibits cell lumen filled with tannin (Rao 1954) (Figs. 3a and 4b). The tomographic images (Fig. 2b, c) illustrate the swelling process after treating the seed in water at 100 °C. Due to the swelling, cavities between seed coat and endosperm disappear and the seed coat cracks open on the inside of the reniform seed where the embryo is located. Looking now closer to that inner cavity the palisade layer becomes thinner up to a certain point where it is interrupted by a small hole of 400 µm in diameter, called chalazal oculus (Gama-Arachchige et al. 2013) (Fig. 3c). In untreated seeds, this opening is filled with the chalazal plug that provides the vascular supply for the embryo (Fig. 3a, b).

## Discussion

### Ecological triggers

Scarification by digestion, as exemplarily shown with elephants, showed only a small albeit significant increase of GR although acid treatment showed better results and therefore let suspect its positive influence on germination (Razanameharizaka et al. 2006; Kempe, Neinhuis and Lautenschläger 2018). The authors that detected a higher GR after acid treatment did not imitate the intestinal scarification process like we did here (HCl of pH 2 and 3 for 1 or 2 h). Studies with HCl treatment for 8 h revealed also a very low GR (Johansson 1999). As a consequence, elephants most probably serve as vectors for seed dispersal (Gerald and Wickens 1982) rather than for breaking seed dormancy. It even seems that digestion can reduce the germination success, visible in the comparison of GR between seeds with or without digestion before heat treatment.

In contrast, not only the treatment with hot water but also the treatment with hot air showed significantly higher GRs. Since the best results were obtained after exposure to a temperature of 75 °C for 40 min and 100 °C for 5 min possible triggers could be high radiation to the savanna soils or wildfires. High solar radiation leads to soil temperatures up to 60 °C, measured in a soil depth of 1.3 cm in North Australia with an equivalent degree of longitude of 14°S (Kalma 1971), or in Niger, 13°N (Vandenbeldt and Williams 1992). In deserts, soil surface temperatures may even exceed 70 °C (Belnap 2003). For northern Angola, we measured soil temperatures of maximum of 32 °C, only during the very cloudy rainy season. For a duration of several weeks or months, this continuous heat might have an influence on the scarification of the seed testa. On the other hand, Kempe et al. (2018) already mentioned the positive impact of savanna fires on germination. Here, the fruit shell of *A. digitata* was shown to serve as a suitable protection of the seeds from the heat generated by savanna wildfires. The moderate heat inside the fruits even improved germination of seeds that were extracted from the fruits. Several authors already discussed the breaking of seed dormancy by means of high temperatures caused by fires. Both, positive impacts on leguminous species (Bradstock and Auld 1995) and negative impacts on weed species (Egley 1990) were observed. Furthermore, studies in Mediterranean fire-prone ecosystems suggest that germination is more successful after the treatment with a single high temperature peak than after a heat treatment simulating summer temperature fluctuations in this area (Moreira and Pausas 2012). Relating to our study, heat generated by fire seems to be the most probable trigger.

### Seed morphometrics

Compared to published data on *A. digitata* seed morphometrics, the Angolan seeds with an average length of 12.0 mm are comparatively large. For Senegal, seeds with a length of only 10.7–11.3 mm (Razanameharizaka et al. 2006) and 10.4–11.4 mm (Niang et al. 2015) were measured. The same applies to the 100-seed dry weight. For the seeds collected in Angola we obtained 53.75 g while Razanameharizaka et al. (2006) recorded a weight of only 39.7 to 42.7 g and Niang et al. (2015) detected 34.15 to 50.59 g. Both studies described the influence of precipitation as well as edapho-climatic environments on seed weight. Accordingly, higher rainfalls cause higher seed weights. Indeed, the annual precipitation in Angola is about 1000 mm while in Senegal it is below 700 mm (Barrientos and Soria 2020). On the other hand, seed size seems to influence germination rate, with smaller seed sizes yielding higher germination rates (Niang et al. 2015). This would explain why in our study, regardless of treatment, GR never exceeded 78% in contrast to various other studies (Esenowo 1991; Johansson 1999; Razanameharizaka et al. 2006; Niang et al. 2015). In addition, our positive control (manual nicking treatment) generated a similar result of 74%. The GR reached in the tests with high temperature therefore correspond with the maximum expected GR. Furthermore, variation within the population or even an individual plant may be important for breaking seed dormancy as well. Seeds of some individuals germinate after heating at low and others at high temperatures (Liyanage and Ooi 2015). This might be another reason for the limited GR in our setups.

### Visualization of testa and germination process

In Malvaceae, to which the baobab belongs, the chalazal slit is known to be capable of preventing water penetration at the stage of dehydration (Egley et al. 1985) but during germination, it probably represents the primary site of water entry, as it was found for various species (Werker 1997; Gama-Arachchige et al. 2013) and could be proven in this study. On the other side, it was observed that after heat treatment, differential thermal expansion separates the macrosclerid cells of the palisade layer, permitting water to penetrate (Rolston 1978). The latter could not be observed here.

Rao (1952) observed a high amount of tannin in between the chalazal cap and the embryo (Figs. 3a and 4b). Tannins are phytochemicals, which exhibit central functions like protection against ultraviolet radiations, pathogens and predators and contribute to physiological functions such as seed maturation and dormancy (Halloin 1982; Shirley 1998; Pourcel et al. 2007). Rao and Deosthale (1982) showed that after soaking seeds in water overnight, 50% of the tannin was lost, while cooking even resulted in a 70% decrease. On the other hand, Harris and Burns (1970) described that high tannin hybrids of *Sorgum* showed a significantly lower seed germination than low tannin hybrids. A correlation between tannin content and germination success therefore can be deduced from these studies. In addition, temperature was mentioned to influence the tannin content. Makkar and Becker (1996) demonstrated that the recovery of tannins decreases with the increase of temperature. Another study found tannin fractions in the seat coat of wheat to be heat labile and described it as naturally occurring inhibitors, which affect the dormancy (Miyamoto, Tolbert and Everson 1961). Adding the results to these former findings on tannin inhibiting effects and its instability with increasing temperature, we presume that *A. digitata* seeds are well adapted to fire-prone savanna areas.

## Conclusion

Our study showed that both, high solar radiation as well as moderate fires are possible triggers for the successful germination of *Adansonia digitata* seeds while the digestive tract of elephants plays an only minor role in removing inhibiting factors. The highest germination rate was achieved with a temperature of 100 °C for 5 min, followed by a treatment with 75 °C for 40 min.

The high amount of tannins in the chalazal region of the seed is considered to be the inhibiting factor of germination. Increasing temperatures lead to a decrease of tannin contents and therefore to germination. The tree is therefore not just well adapted to fire-prone savannas but the success of germination depends strongly on heat, provoked by wildfires or prolonged exposition to the sun.

## Supplementary information

**Additional file 1: Figure S1.** Relative germination rate in percentage of *Adansonia digitata* seeds treated with acids and digested by elephants. Observation time: 10 weeks (W). Control experiments marked in green. **Figure S2.** Relative germination rate in percentage of *Adansonia digitata* seeds after digestion of elephants and exposed to heat for 5 min. Observation time: 10 weeks (W). Control experiments marked in green. **Figure S3.** Relative germination rate in percentage of *Adansonia digitata* seeds exposed to heat for 5 min. Observation time: 10 weeks (W). Control experiments marked in green. **Figure S4**. Relative germination rate in percentage of *Adansonia digitata* seeds exposed to heat for 40 min. Observation time: 10 weeks (W). Control experiments marked in green.

## Data Availability

All data are available from the corresponding author.

## Abbreviations

A: *Adansonia*
C: Celsius
GR: Germination rate
H_2_SO_4_: Sulfuric acid
HCl: Hydrochlorid acid
N: North
S: South
SEM: Scanning electron microscope
