# Supplementary material for: Adansonia digitata germination tests. Elephants or heat: what causes scarification of seed to facilitate germination?
Source: Bot Stud. 2020 Jun 16;61:19. doi: 10.1186/s40529-020-00296-0 (PMC7297922; doi:10.1186/s40529-020-00296-0)
Supplement: Supplementary file 1 — Additional file 1: Figure S1. Relative germination rate in percentage of Adansonia digitata seeds treated with acids and digested by elephants. Observation time: 10 weeks (W). Control experiments marked in green. Figure S2. Relative germination rate in percentage of Adansonia digitata seeds after digestion of elephants and exposed to heat for 5 min. Observation time: 10 weeks (W). Control experiments marked in green. Figure S3. Relative germination rate in percentage of Adansonia digitata seeds exposed to heat for 5 min. Observation time: 10 weeks (W). Control experiments marked in green. Figure S4. Relative germination rate in percentage of Adansonia digitata seeds exposed to heat for 40 min. Observation time: 10 weeks (W). Control experiments marked in green. [file 40529_2020_296_MOESM1_ESM.docx]

**Additional file 1**

Figure S1: Relative germination rate in percentage of *Adansonia digitata* seeds treated with acids and digested by elephants. Observation time: 10 weeks (W). No treatment (*green*) as a control.

Most of seeds treated with H_2_SO_4_, HCl or digested by elephants germinated within the third and fourth week. In this period maximum of GR was reached by H_2_SO_4_ for 15 min (12%) and also the test sets of HCl and elephants digestion achieved its peaks. In week 5-6 germination starts to slow down and just 3.3% of treated seeds germinate in week 7-8.

Figure S2 Relative germination rate in percentage of *Adansonia digitata* seeds after digestion of elephants and exposed to heat for 5 minutes. Observation time: 10 weeks (W). Control experiments marked in green.

GR of digested seeds germinated in soil or faeces of elephants is quite low compared to seeds with additional heat treatment. However, in all test sets the GR increase to a peak in week 3-4 and sink down slowly afterwards. Like in short-time heat treatment the maximum of GR achieves 100 °C for 5minutes followed by 75 °C

Figure S3: Relative germination rate in percentage of *Adansonia digitata* seeds exposed to heat for 5 minutes. Observation time: 10 weeks (W). Control experiments marked in green.

The germination after short-time heat treatment is nearly equal distributed between week 3-4 (46%) and week 5-6 (49%). While 5 minutes of 75 °C reached its peak in week 3-4, 100 °C for 5 minutes has a maximum of 42% in week 5-6. Germination after heat treatment over 100 °C is very low because the embryo could already be killed.

Figure S4 Relative germination rate in percentage of *Adansonia digitata* seeds exposed to heat for 40 minutes. Observation time: 10 weeks (W). Control experiments marked in green.

After treatments in hot water or wet hot sand for 40 minutes rapid germination occurred in week 3 and 4 while it was delayed by two weeks after exposure to dry hot sand. In 2018, 95% of the germinated seeds treated with hot water germinated in week 3-4, 64% of those treated with wet sand (75 °C), but just 30% of the seed treated in dry heat (75 °C). The latter showed the main germination peak in week 5-6 (70%). At lower temperature (50 °C) this trend slows down but is still significant (P<0.01).
